# Supplementary material for: Molecular Epidemiology of Antibiotic Resistance Genes and Virulence Factors in Multidrug-Resistant Escherichia coli Isolated from Rodents, Humans, Chicken, and Household Soils in Karatu, Northern Tanzania
Source: Int J Environ Res Public Health. 2022 Apr 28;19(9):5388. doi: 10.3390/ijerph19095388 (PMC9102629; doi:10.3390/ijerph19095388)
Supplement: Supplementary file 1 [file ijerph-19-05388-s001.zip › ijerph-1603695-supplementary.pdf]

Table S1. List of primers used for amplification of selected antimicrobial resistance and virulence genes of *E. coli* isolates.

Target Gene	Direction	Primer Sequence (5′-3′)	Annealing temp (°C)	Amplicon size (bp)	References
blaIMP	Forward	TCGTTTGAAGAAGTTAACG	60	568	[31]
	Reverse	ATGTAAGTTTCAAGAGTGATGC			
blaVIM	Forward	GGTGTTTGGTCGCATATCGCAA	60	502	[31]
	Reverse	ATTCAGCCAGATCGGCATCGGC			
blaNDM	Forward	GGTTTGGCGATCTGGTTTTC	60	624	[31]
	Reverse	CGGAATGGCTCATCACGATC			
blaKPC	Forward	CATTCAAGGGCTTTCTTGCTGC	60	498	[31]
	Reverse	ACGACGGCATAGTCATTTGC			
blaOXA-48	Forward	TTCTGTTGTTTGGGTTTCGC	55	190	[32]
	Reverse	ACGCAGGAATTGAATTTGTTC			
tet(A)	Forward	GGTTCACTCGAACGACGTCA	56	577	[33]
	Reverse	CTGTCCGACAAGTTGCATGA			
tet(B)	Forward	CCTCAGCTTCTCAACGCGTG	56	634	[33]
	Reverse	GCACCTTGCTCATGACTCTT			
blaCTX-M	Forward	ATGTGCAGYACCAGTAARGTKATGGC	50	593	[34]
	Reverse	TGGGTRAARTARGTSACCAGAAYCAGCGG			
blaTEM	Forward	GTCGCCGCATACACTATTCTCA	68	258	[32]
	Reverse	CGCTCGTCGTTTGGTATGG			
blaSHV	Forward	GCCTTGACCGCTGGGAAAC	68	319	[32]
	Reverse	GGCGTATCCCGCAGATAAAT			
eaeA	Forward	ATGCTTAGTGCTGGTTTAGG	58	248	[35]
	Reverse	GCCTTCATCATTTCGCTTTC			
east	Forward	CACAGTATATCCGAAGGC	50	97	[36]
	Reverse	CGAGTGACGGCTTTGTAG			
hlyA	Forward	AACAAGGATAAGCACTGTTCTGGCT	63	1177	[37]
	Reverse	ACCATATAAGCGGTCATTCCCGTCA			
ompA	Forward	AGCTATCGCGATTGCAGTG	52	919	[38]
	Reverse	GGTGTTGCCAGTAACCGG			
traT	Forward	GGTGTGGTGCGATGAGCACAG	63	290	[39]
	Reverse	CACGGTTCAGCCATCCCTGAG			
cnf1/2	Forward	TCGTTATAAAATCAAACAGTG	51	446	[40]

	Reverse	CTTTACAATATTGACATGCTG			
bfp	Forward	AATGGTGCTTGCGCTTGCTGC	56	324	[41]
	Reverse	GCCGCTTTATCCAACCTGGTA			
stx1	Forward	TCTCAGTGGGCGTTCTTATG	58	388	[35]
	Reverse	TACCCCCTCAACTGCTAATA			

Table S2. Multiplex PCR conditions used during amplification of antibiotic resistance and virulence genes of MDR *E. coli* isolates.

Program	Targeted Genes	Amplification conditions for resistance genes						Number of cycles
		**Initial Denaturation**	**Denaturation**	**Annealing**	**Primer extension**	**Final extension**	**Amplicon size (bp)**	
	blaIMP	95 ^{0}C for 5 min	95 ^{0}C for 30 sec	60 ^{0}C for 30 sec	72 ^{0}C for 1 min	72 ^{0}C for 7 min	568	x35
**PCR 1**	*blaVIM*	95 ^{0}C for 5 min	95 ^{0}C for 30 sec	60 ^{0}C for 30 sec	72 ^{0}C for 1 min	72 ^{0}C for 7 min	502	x35
	blaNDM-1	95 ^{0}C for 5 min	95 ^{0}C for 30 sec	60 ^{0}C for 30 sec	72 ^{0}C for 1 min	72 ^{0}C for 7 min	624	x35
	qnrB	95 ^{0}C for 5 min	95 ^{0}C for 30 sec	60 ^{0}C for 30 sec	72 ^{0}C for 1 min	72 ^{0}C for 7 min	264	x35
	qnrA	95 ^{0}C for 5 min	95 ^{0}C for 30 sec	60 ^{0}C for 30 sec	72 ^{0}C for 1 min	72 ^{0}C for 7 min	605	x35
	tetA	95 ^{0}C for 5 min	94 ^{0}C for 45 sec	55 ^{0}C for 30 sec	72 ^{0}C for 1 min	72 ^{0}C for 10 min	577	x30
**PCR 2**	*tetB*	95 ^{0}C for 5 min	94 ^{0}C for 45 sec	55 ^{0}C for 30 sec	72 ^{0}C for 1 min	72 ^{0}C for 10 min	637	x30
	blaSHV	95 ^{0}C for 5 min	94 ^{0}C for 45 sec	55 ^{0}C for 30 sec	72 ^{0}C for 1 min	72 ^{0}C for 10 min	319	x30
	blaTEM	95 ^{0}C for 5 min	94 ^{0}C for 45 sec	55 ^{0}C for 30 sec	72 ^{0}C for 1 min	72 ^{0}C for 10 min	258	x30
	blaCTX-M	95 ^{0}C for 5 min	94 ^{0}C for 45 sec	55 ^{0}C for 30 sec	72 ^{0}C for 1 min	72 ^{0}C for 10 min	593	x35
**PCR 3**	*blaKPC*	95 ^{0}C for 5 min	94 ^{0}C for 45 sec	55 ^{0}C for 30 sec	72 ^{0}C for 1 min	72 ^{0}C for 10 min	498	x35
	blaOXA-48	95 ^{0}C for 5 min	94 ^{0}C for 45 sec	55 ^{0}C for 30 sec	72 ^{0}C for 1 min	72 ^{0}C for 10 min	238	x35
	east	94 ^{0}C for 5 min	94 ^{0}C for 1 min	58 ^{0}C for 30 sec	68 ^{0}C for3 min	72 ^{0}C for10min	97	x30
**PCR 4**	*ompA*	94 ^{0}C for 5 min	94 ^{0}C for 1 min	58 ^{0}C for 30 sec	68 ^{0}C for 3 min	72 ^{0}C for 10 min	919	x30
	cnf	94 ^{0}C for 5 min	94 ^{0}C for 1 min	58 ^{0}C for 30 sec	68 ^{0}C for 3 min	72 ^{0}C for 10 min	446	x30
	traT	94 ^{0}C for 5 min	94 ^{0}C for 1 min	58 ^{0}C for 30 sec	68 ^{0}C for 3 min	72 ^{0}C for 10 min	290	x30
	bfp	95 ^{0}C for 5 min	95 ^{0}C for 30sec	55 ^{0}C for 30 sec	72 ^{0}C for 3 min	72 ^{0}C for 7 min	324	x35
**PCR 5**	*stx-1*	95 ^{0}C for 5min	95 ^{0}C for 30 sec	55 ^{0}C for 30 sec	72 ^{0}C for 3 min	72 ^{0}C for 7 min	338	x35
	eae	95 ^{0}C for 5 min	95 ^{0}C for 30 sec	55 ^{0}C for 30 sec	72 ^{0}C for 3 min	72 ^{0}C for 7 min	248	x35
	hlyA	95 ^{0}C for 5 min	95 ^{0}C for 30 sec	55 ^{0}C for 30 sec	72 ^{0}C for 3 min	72 ^{0}C for 7 min	1177	x35
